# Targeting ADAR1 Restores Interferon Signaling and Enhances Immunotherapy Response in Multiple Myeloma

**DOI:** 10.3390/ijms27156602

**Published:** 2026-07-24

**Authors:** Songze Leng, Yaoyao Tian, Yao Liu, Weiwei Zhao, Wei Wang

**Affiliations:** 1 Department of Hematology, Second Affiliated Hospital of Harbin Medical University, Harbin 150001, China; 2 Department of Cell Biology, School of Basic Medicine, Harbin Medical University, Harbin 150081, China

**Keywords:** multiple myeloma, ADAR1, IFNα, MDA5, CD8^+^ T cells

## Abstract

Multiple myeloma (MM) remains an incurable hematological malignancy in which tumor-intrinsic immune evasion limits the efficacy of immunotherapy. Here, we identify the RNA editing enzyme ADAR1 as a key regulator of innate immune suppression in MM. Integrative analyses of bulk and single-cell transcriptomic datasets, together with clinical validation, demonstrated that ADAR1 is upregulated in malignant plasma cells and is associated with adverse clinical outcomes and reduced CD8^+^ T-cell infiltration. Mechanistically, ADAR1 knockdown increased the association of endogenous dsRNA with melanoma differentiation-associated protein 5 (MDA5), restoring type I interferon (IFN) signaling, enhancing IFNα production and STAT1 activation, and promoting CD8^+^ T-cell proliferation and cytotoxic function. These effects were largely abolished by MDA5 depletion, establishing a functional ADAR1–MDA5 signaling axis in MM. In vivo, treatment with 8-azaadenosine significantly potentiated the antitumor efficacy of PD-1 blockade, resulting in reduced tumor growth, increased tumor cell apoptosis, elevated IFNα expression, and enhanced CD8^+^ T-cell infiltration. Together, our findings demonstrate that ADAR1-mediated RNA editing enables immune evasion by restricting MDA5-dependent sensing of endogenous dsRNA and highlight the ADAR1–MDA5-type I interferon axis as a promising therapeutic target for improving immunotherapy in multiple myeloma.

## 1. Introduction

Multiple myeloma (MM) is a hematological malignancy characterized by the clonal expansion of malignant plasma cells within the bone marrow (BM) [1]. Over the past decade, the introduction of proteasome inhibitors, immunomodulatory agents, monoclonal antibodies, and chimeric antigen receptor (CAR) T-cell therapy has significantly improved patient outcomes [2]. Nevertheless, MM remains largely incurable, as most patients ultimately relapse and develop resistance to current treatments, including immunotherapies [3,4]. Elucidating the molecular mechanisms that underlie disease progression and therapeutic resistance is therefore essential for identifying effective targets for intervention.

The bone marrow microenvironment plays a central role in MM pathogenesis and treatment response. It comprises malignant plasma cells, stromal components, and diverse immune populations, including T cells, natural killer (NK) cells, dendritic cells, and macrophages. These elements interact through complex signaling networks to establish an immunoregulatory niche that can either support or restrain tumor growth [5,6]. Among immune effectors, CD8^+^ cytotoxic T lymphocytes (CTLs) are critical mediators of antitumor immunity. However, in MM, CD8^+^ T cells commonly exhibit reduced infiltration and impaired effector function, accompanied by sustained antigen exposure and upregulation of inhibitory pathways [7,8,9]. This dysfunctional state contributes to immune escape and limits the efficacy of immunotherapeutic strategies.

Tumor-intrinsic mechanisms that actively shape this immunosuppressive environment remain incompletely defined. Recent advances in high-throughput sequencing and integrative bioinformatics have enabled systematic dissection of tumor–immune interactions, facilitating the identification of key regulatory molecules that link intrinsic tumor biology with immune dysfunction [8,10]. In particular, pathways involved in innate immune sensing and type I interferon (IFN) signaling have emerged as critical determinants of antitumor immunity and responsiveness to immune checkpoint blockade [10,11].

Adenosine deaminase acting on RNA 1 (ADAR1) is an RNA editing enzyme that catalyzes adenosine-to-inosine (A-to-I) conversion in double-stranded RNA (dsRNA), thereby modulating the recognition of endogenous RNA by innate immune sensors such as melanoma differentiation–associated protein 5 (MDA5) [12,13]. Through this mechanism, ADAR1 can attenuate type I IFN signaling and enable tumor cells to evade immune surveillance [13,14]. While ADAR1 has been implicated in immune regulation and resistance to immunotherapy in solid tumors, its functional role and mechanistic relevance in MM remain poorly understood.

In this study, we performed an integrative analysis of transcriptomic datasets, immune infiltration profiles, and clinical outcomes to identify regulators associated with CD8^+^ T-cell dysfunction in MM. We identify ADAR1 as a key tumor-intrinsic factor that is upregulated in malignant plasma cells and negatively associated with CD8^+^ T-cell infiltration. Mechanistically, we demonstrate that ADAR1 suppresses MDA5-dependent type I IFN signaling, thereby limiting CD8^+^ T-cell proliferation and cytotoxic activity. Furthermore, targeting ADAR1 enhances interferon responses and improves sensitivity to PD-1 blockade in vitro and in vivo. These findings define an ADAR1–MDA5–IFNα regulatory axis that links RNA editing to immune evasion in MM and highlight ADAR1 as a potential therapeutic target to enhance immunotherapy efficacy.

## 2. Results

### 2.1. Immune Landscape of Multiple Myeloma and Identification of Candidate Regulators

**Figure 1:**
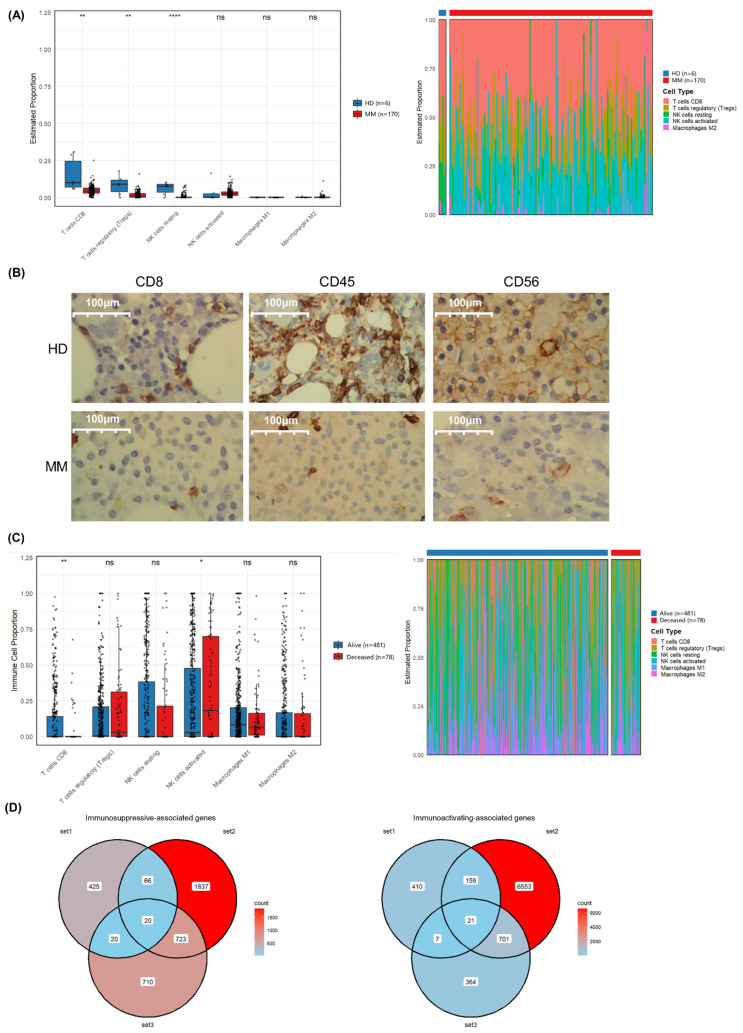
Characterization of the immune microenvironment in multiple myeloma and identification of candidate immune regulatory genes. (**A**) Immune cell infiltration analysis using the CIBERSORT algorithm in GEO transcriptomic datasets. Box plots compare the distributions of six representative immune cell populations between multiple myeloma patients and healthy donors, while stacked bar plots show the relative proportions of these immune cell populations in individual bone marrow samples. (**B**) Immunohistochemical staining of bone marrow tissues from healthy donors (HDs) and MM patients. (**C**) Comparison of 6 representative immune cell infiltration patterns between patients with favorable survival outcomes and those with poor prognosis. (**D**) Venn diagrams showing the intersection of three gene sets: genes correlated with CD8^+^ T-cell infiltration (Spearman correlation analysis), DEGs between MM and healthy samples, and genes associated with OS. ns, not significant; * *p* < 0.05; ** *p* < 0.01; **** *p* < 0.0001.

To characterize the immune microenvironment of MM, we analyzed immune cell infiltration in publicly available transcriptomic datasets using the CIBERSORT algorithm. We focused on six immune cell populations with established relevance to antitumor immunity and MM pathogenesis, including CD8^+^ T cells, regulatory T cells (Tregs), resting NK cells, activated NK cells, M1 macrophages, and M2 macrophages. Compared with healthy donors (HDs), MM bone marrow samples exhibited significantly reduced infiltration of CD8^+^ T cells and NK cells, accompanied by alterations in macrophage and Treg populations, indicating a broadly dysregulated immune landscape (Figure 1A). Immunohistochemical analysis of clinical bone marrow specimens further confirmed a marked reduction in CD8^+^ T-cell infiltration in MM patients (Figure 1B).

We next examined the clinical relevance of immune infiltration. Stratification by OS demonstrated that patients with poor outcomes exhibited significantly lower CD8^+^ T-cell infiltration than those with favorable prognosis (Figure 1C), indicating a link between impaired cytotoxic immunity and disease progression.

To identify molecular regulators associated with CD8^+^ T-cell dysfunction, we performed an integrative analysis combining (i) genes correlated with CD8^+^ T-cell infiltration, (ii) DEGs between MM and healthy samples, and (iii) genes associated with OS. This approach yielded 42 candidate genes, including 20 upregulated genes negatively associated with CD8^+^ T-cell infiltration and poor survival, and 22 downregulated genes positively associated with immune infiltration and favorable prognosis (Figure 1D). Among these, the RNA editing enzyme ADAR1 was prioritized for further investigation.

### 2.2. ADAR1 Is Upregulated in Multiple Myeloma and Correlates with Poor Clinical Outcomes

**Figure 2:**
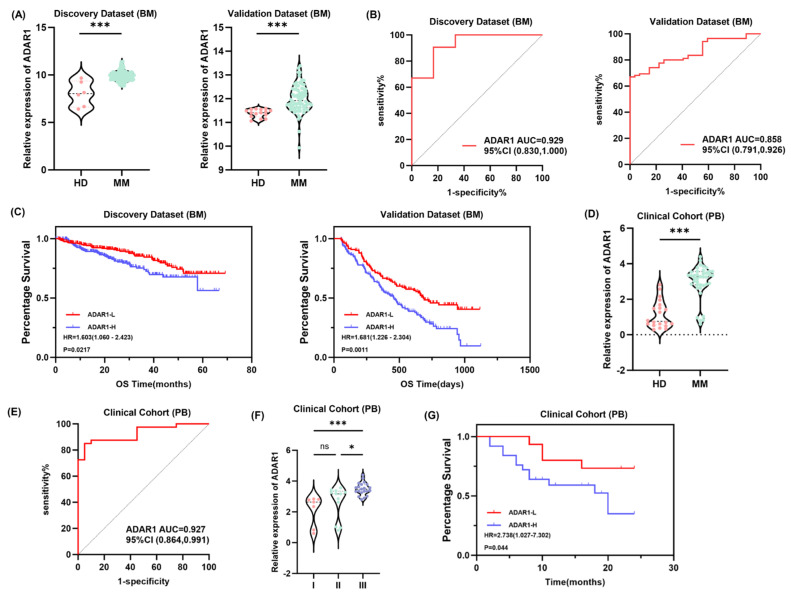
Expression pattern and clinical significance of ADAR1 in multiple myeloma. (**A**) ADAR1 expression in the discovery dataset and independent validation dataset of publicly available bone marrow transcriptomic datasets. (**B**) ROC curve analysis evaluating the diagnostic performance of ADAR1 in distinguishing multiple myeloma patients from healthy donors in the discovery and validation datasets. (**C**) Kaplan–Meier OS analysis stratified by ADAR1 expression in the discovery and validationdatasets. (**D**) Comparison of ADAR1 expression levels in peripheral blood samples from MM patients and healthy donors in the clinical cohort. (**E**) ROC curve analysis evaluating the diagnostic value of ADAR1 expression in peripheral blood samples. (**F**) ADAR1 expression across different R-ISS stages in MM patients. (**G**) Kaplan–Meier OS analysis based on ADAR1 expression levels in the clinical cohort. ns, not significant; * *p* < 0.05; *** *p* < 0.001.

We next assessed the clinical relevance of ADAR1 expression. Analysis of transcriptomic datasets demonstrated that ADAR1 expression was significantly elevated in MM samples compared with healthy donors in both the discovery and validation bone marrow dataset (Figure 2A). ROC analysis demonstrated good diagnostic performance for distinguishing MM from healthy controls (Figure 2B). Moreover, high ADAR1 expression was associated with significantly shorter overall survival (OS) in both cohorts (Figure 2C).

To further validate these findings, we analyzed an independent peripheral blood (PB) cohort. Consistent with the bone marrow transcriptomic results, ADAR1 expression was markedly increased in MM patients compared with healthy controls (Figure 2D) and showed strong diagnostic accuracy in distinguishing disease status (Figure 2E). Notably, when stratified by R-ISS stage, ADAR1 expression did not differ significantly between stage I and II patients; however, a significant increase was observed in stage III patients compared with both stage I and II groups (Figure 2F). Furthermore, higher ADAR1 expression was associated with significantly worse survival outcomes in this cohort (Figure 2G).

Collectively, these findings demonstrate that ADAR1 is consistently upregulated in MM across independent cohorts and sample types, is associated with disease severity, and correlates with poor prognosis, supporting its clinical relevance as a potential biomarker for disease progression and outcome prediction in MM.

### 2.3. ADAR1 Expression Inversely Correlates with CD8^+^ T-Cell Infiltration

**Figure 3:**
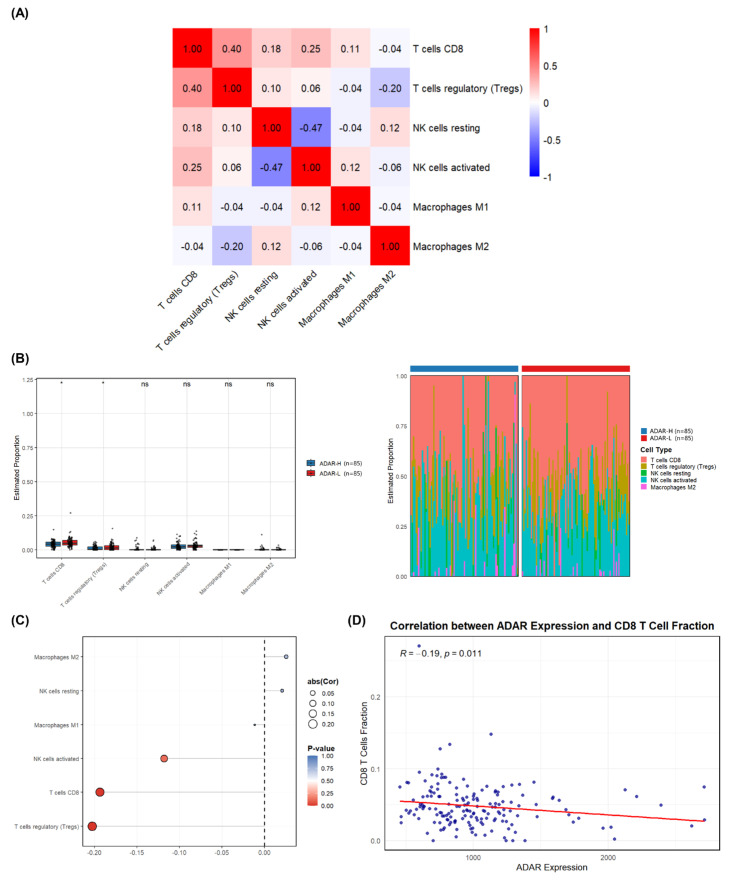
Association between ADAR1 expression and immune cell infiltration in multiple myeloma. (**A**) Correlation heatmap of six representative immune cell populations in MM samples based on CIBERSORT analysis. (**B**) Comparison of immune cell infiltration between ADAR1-high and ADAR1-low groups stratified by the median ADAR1 expression level. Box plots compare the distributions of six representative immune cell populations, and stacked bar plots show their relative proportions in individual samples. (**C**) Correlation analysis between ADAR1 expression and the infiltration levels of six representative immune cell populations. (**D**) Scatter plot showing the negative correlation between ADAR1 expression and CD8^+^ T-cell infiltration. ns, not significant; * *p* < 0.05.

Given the association between CD8^+^ T-cell deficiency and poor prognosis, we next investigated the relationship between ADAR1 expression and immune cell infiltration. Correlation analysis revealed distinct interaction patterns among six representative immune cell populations in the MM microenvironment (Figure 3A).

MM samples were subsequently stratified according to the median ADAR1 expression level. Compared with the ADAR1-high group, the ADAR1-low group exhibited increased CD8^+^ T-cell infiltration (Figure 3B). Consistently, correlation analysis demonstrated that ADAR1 expression was negatively associated with CD8^+^ T-cell abundance (Figure 3C). This inverse relationship was further confirmed by Spearman correlation analysis (Figure 3D). These findings suggest that elevated ADAR1 expression is linked to impaired CD8^+^ T-cell infiltration.

### 2.4. ADAR1 Is Enriched in Malignant Plasma Cells and Associated with Impaired Interferon Signaling

**Figure 4:**
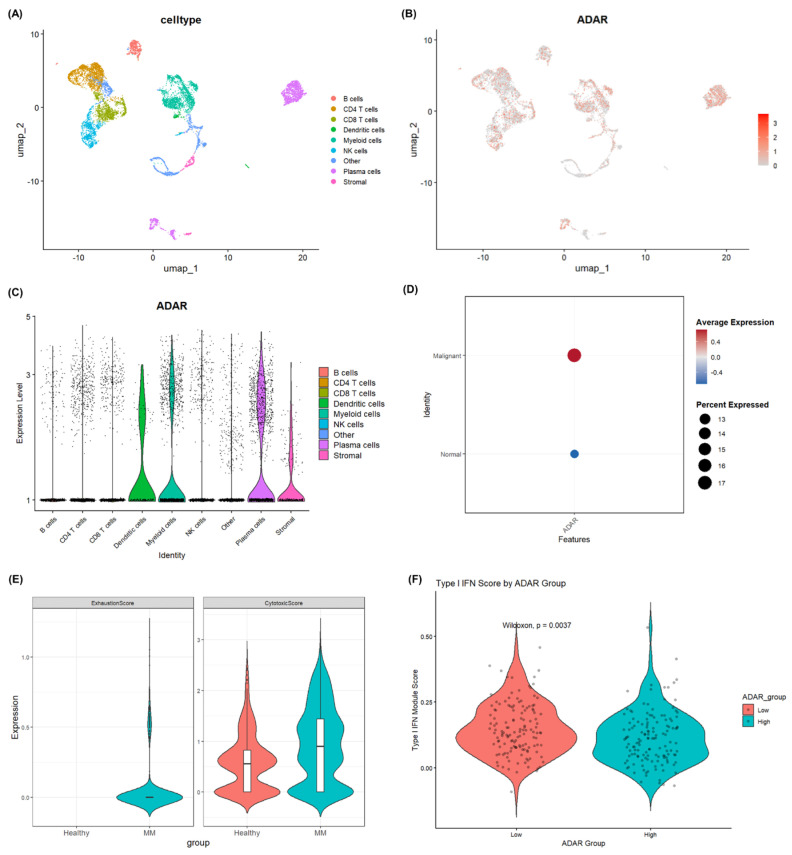
Single-cell transcriptomic analysis showing ADAR1 expression patterns and its association with IFN signaling in multiple myeloma. (**A**) UMAP visualization of single-cell RNA-seq data from the GSE124310 dataset showing major cell populations in the MM bone marrow microenvironment. (**B**) Feature plot showing the distribution of ADAR1 expression across different cell clusters. (**C**) Violin plot comparing ADAR1 expression levels among different immune cell populations. (**D**) Comparison of ADAR1 expression between malignant plasma cells and plasma cells from healthy donors. (**E**) Analysis of T-cell exhaustion signatures in MM samples. (**F**) Type I IFN pathway activity in malignant plasma cells stratified by ADAR1 expression levels.

To determine the cellular origin of ADAR1, we analyzed single-cell RNA sequencing data from MM bone marrow. UMAP clustering identified major cellular populations, including plasma cells, T cells, B cells, and myeloid cells (Figure 4A). ADAR1 expression was predominantly localized to plasma cells (Figure 4B), and was significantly higher in this compartment compared with other immune populations (Figure 4C).

Importantly, ADAR1 expression was markedly increased in malignant plasma cells relative to normal counterparts (Figure 4D). In parallel, MM samples exhibited enhanced T-cell exhaustion signatures (Figure 4E). Functional pathway analysis demonstrated significantly lower type I interferon signaling activity in ADAR-high cells than in ADAR-low cells (Figure 4F). Although the distributions partially overlapped, the difference remained statistically significant, suggesting that elevated ADAR1 expression is associated with suppression of interferon signaling in MM.

### 2.5. ADAR1 Suppresses MDA5-Dependent Type I Interferon Signaling

**Figure 5:**
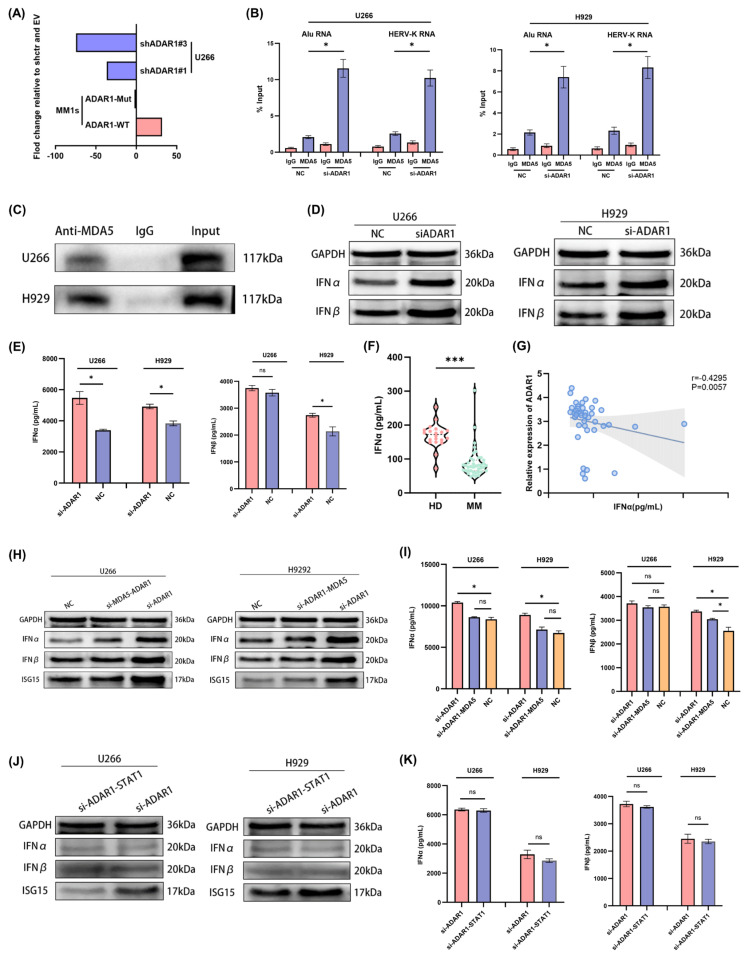
ADAR1 suppresses MDA5-mediated type I interferon signaling in multiple myeloma. (**A**) Comparison of A-to-G RNA editing frequency between ADAR1-high and ADAR1-low samples in the GSE110486 dataset. (**B**) RIP-qPCR analysis showing the enrichment of Alu dsRNA associated with ADAR1 and MDA5 following ADAR1 knockdown. (**C**) Western blot validation of MDA5 immunoprecipitation in the RIP assay. (**D**) Western blot analysis of IFNα and IFNβ expression after ADAR1 knockdown in U266 and H929 cells co-cultured with HS-5 stromal cells. (**E**) ELISA measurement of IFNα and IFNβ secretion following ADAR1 knockdown. (**F**) Comparison of IFNα levels in peripheral blood samples from healthy donors and MM patients. (**G**) Spearman correlation analysis between ADAR1 expression and IFNα levels in MM patient samples. (**H**) Western blot analysis of IFNα, IFNβ, and ISG15 expression following simultaneous knockdown of ADAR1 and MDA5. (**I**) ELISA measurement of IFNα and IFNβ secretion after co-silencing ADAR1 and MDA5. (**J**) Western blot analysis of ISG15 expression following combined knockdown of ADAR1 and STAT1. (**K**) ELISA analysis of IFNα and IFNβ secretion after STAT1 knockdown. ns, not significant; * *p* < 0.05; *** *p* < 0.001.

To investigate the mechanistic role of ADAR1, we first assessed its RNA editing activity. Analysis of the GSE110486 dataset showed that A-to-G RNA editing frequency was significantly elevated in ADAR1-high samples (Figure 5A), consistent with enhanced ADAR1 enzymatic activity.

To further examine the relationship between ADAR1 and MDA5, RNA immunoprecipitation (RIP) assays were performed. RIP-qPCR showed that ADAR1 knockdown significantly increased the enrichment of Alu dsRNA associated with MDA5 (Figure 5B). Successful immunoprecipitation of MDA5 was confirmed by Western blot analysis (Figure 5C).

Functional experiments in MM cell lines co-cultured with stromal cells demonstrated that ADAR1 knockdown markedly increased IFNα expression and secretion, whereas IFNβ levels remained largely unchanged (Figure 5D,E). Consistently, IFNα levels were reduced in MM patient samples and inversely correlated with ADAR1 expression (Figure 5F,G).

To determine whether MDA5 mediates this effect, we performed co-silencing ex-periments. Simultaneous knockdown of ADAR1 and MDA5 abrogated the induction of IFNα, IFNβ, and ISG15 observed following ADAR1 depletion (Figure 5H,I), indicating that MDA5 is required for ADAR1-regulated IFN signaling. In contrast, STAT1 knock-down did not affect IFNα or IFNβ levels but reduced ISG15 expression (Figure 5J,K), supporting its role as a downstream effector. These results define a functional ADAR1–MDA5–IFN signaling axis in MM.

### 2.6. The ADAR1–MDA5–IFNα Axis Regulates CD8^+^ T-Cell Function and Immunotherapy Response

**Figure 6:**
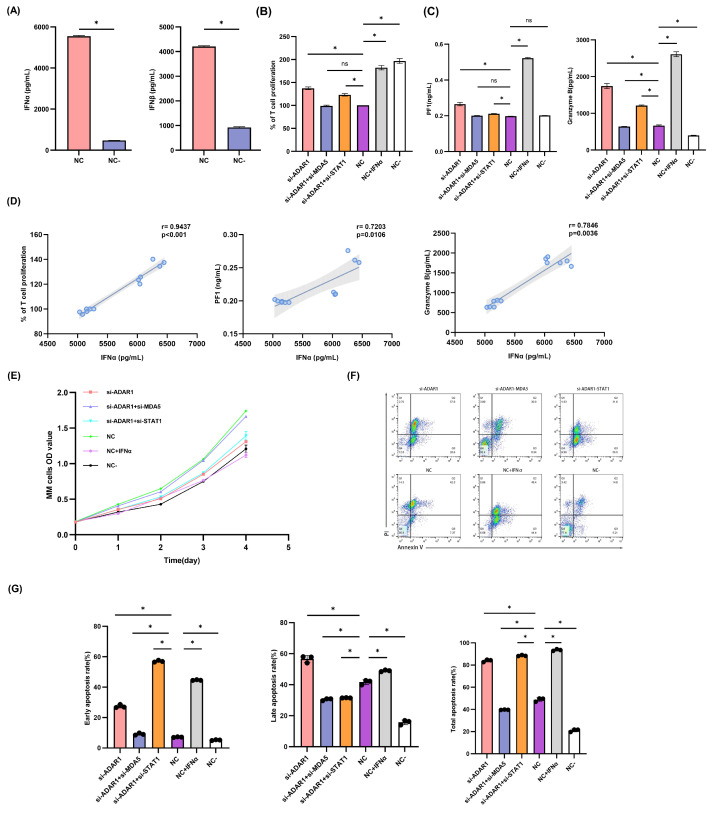
The ADAR1–MDA5–IFNα axis regulates CD8^+^ T-cell activity and affects the response of multiple myeloma cells to PD-1 blockade. (**A**) ELISA analysis of IFNα and IFNβ secretion in U266 monoculture and U266–HS-5 co-culture systems. (**B**) CCK-8 assay measuring the relative CD8^+^ T-cell proliferation in the triple co-culture system. T-cell proliferation in the NC group was normalized to 100%, and values in the remaining groups are presented relative to the NC control. (**C**) ELISA analysis of PF1 and GZMB secretion in the triple co-culture system. (**D**) Spearman correlation analysis between IFNα levels and CD8^+^ T-cell proliferation and cytotoxic molecule expression. (**E**) CCK-8 assay evaluating U266 cell proliferation under different experimental conditions. (**F**) Flow cytometric analysis of U266 cell apoptosis using Annexin V/PI staining. (**G**) Flow cytometric analysis of U266 cell apoptosis, including early, late, and total apoptosis. ns, not significant; * *p* < 0.05.

We next examined the functional consequences of ADAR1-mediated IFN suppression within the tumor microenvironment. Co-culture of MM cells with stromal cells significantly increased IFNα secretion compared with monoculture, while IFNβ levels remained unchanged (Figure 6A), indicating microenvironmental modulation of IFN responses.

In a triple co-culture system incorporating MM cells, stromal cells, and CD8^+^ T cells, ADAR1 knockdown significantly enhanced CD8^+^ T-cell proliferation. This effect was recapitulated by exogenous IFNα treatment and was abolished by MDA5 co-silencing (Figure 6B). Similarly, secretion of cytotoxic mediators perforin and granzyme B was increased following ADAR1 depletion or IFNα stimulation (Figure 6C), and positively correlated with IFNα levels (Figure 6D).

Functionally, MM cell proliferation was highest under control conditions and reduced upon activation of IFN signaling (Figure 6E). Following PD-1 blockade, ADAR1 silencing or IFNα treatment significantly increased MM cell apoptosis, characterized by higher early and total apoptotic fractions. In contrast, MDA5 knockdown attenuated, whereas STAT1 silencing reversed, the pro-apoptotic effects of ADAR1 depletion (Figure 6F,G). These findings indicate that ADAR1 restricts antitumor immunity by suppressing IFN-driven CD8^+^ T-cell activity and limiting responsiveness to immune checkpoint inhibition.

### 2.7. ADAR1 Inhibition Enhances the Efficacy of PD-1 Blockade In Vivo

**Figure 7:**
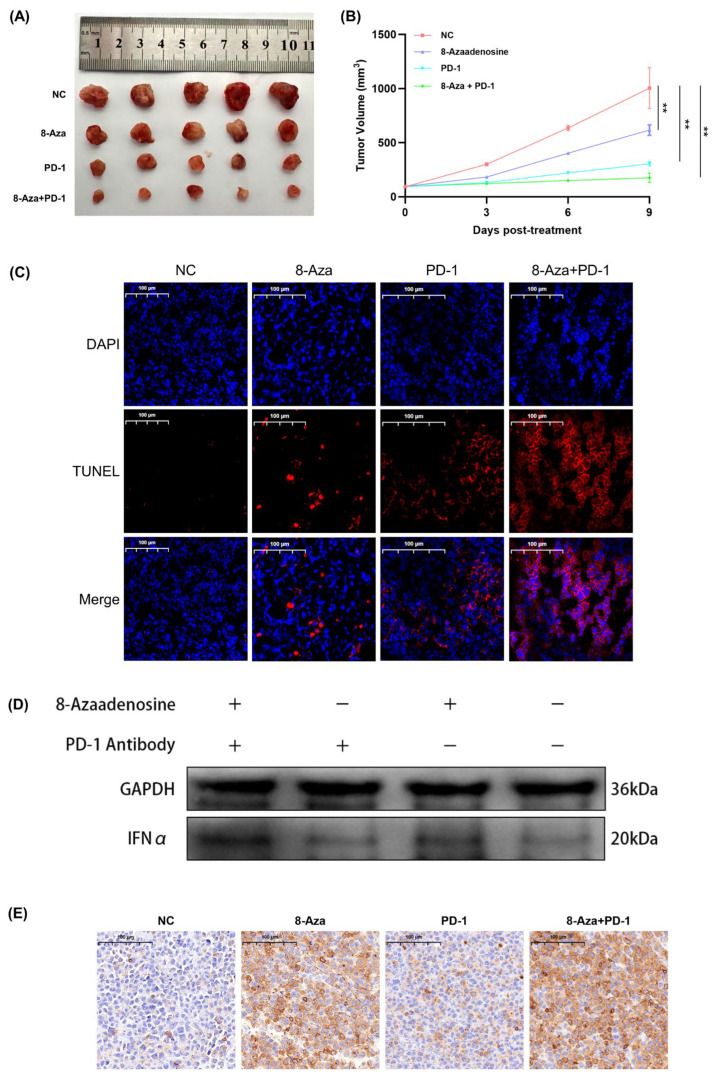
ADAR1 inhibition enhances the antitumor efficacy of PD-1 blockade in a multiple myeloma mouse model. (**A**) Endpoint tumor volumes in the MOPC315/BALB/c mouse model across different treatment groups. (**B**) Tumor growth curves of mice treated with NC, 8-azaadenosine, PD-1 blockade, or combination therapy. (**C**) Representative TUNEL staining of tumor sections showing apoptotic cells (red) with DAPI nuclear counterstaining (blue). (**D**) Western blot analysis of IFNα protein expression in tumor tissues. (**E**) Immunohistochemical staining showing CD8^+^ T-cell infiltration in tumor tissues. ** *p* < 0.01.

To evaluate the therapeutic relevance of ADAR1 targeting, we established an immunocompetent MOPC315/BALB/c mouse model. Mice were treated with vehicle control, 8-azaadenosine, a pharmacological agent targeting ADAR1, PD-1 blockade, or the combination of both. While either monotherapy modestly reduced tumor growth, combination treatment produced a significantly greater suppression of tumor progression (Figure 7A,B).

TUNEL staining showed increased apoptosis in tumors following 8-azaadenosine or PD-1 blockade treatment, with the strongest effect observed in the combination group (Figure 7C). IFNα expression was also increased in tumor tissues, particularly after combination treatment (Figure 7D). In parallel, immunohistochemical staining demonstrated enhanced CD8^+^ T-cell infiltration in tumors treated with 8-azaadenosine alone or in combination with PD-1 blockade (Figure 7E). These findings support the therapeutic potential of targeting ADAR1 to enhance the antitumor efficacy of PD-1 blockade in MM.

## 3. Discussion

MM remains an incurable hematological malignancy despite substantial therapeutic advances. Although proteasome inhibitors, immunomodulatory agents, monoclonal antibodies, and cellular immunotherapies have significantly improved patient outcomes, most patients ultimately relapse or develop treatment resistance [15,16]. Increasing evidence indicates that tumor-intrinsic mechanisms, in concert with the bone marrow microenvironment, play a critical role in immune evasion and therapeutic failure [17,18]. In particular, impaired infiltration and functional exhaustion of CD8^+^ cytotoxic T cells represent key features of immune dysfunction in MM, yet the molecular drivers of this process remain incompletely defined [19].

In the present study, we identify ADAR1 as a key tumor-intrinsic regulator of immune evasion in MM. Integrative analyses of bulk and single-cell transcriptomic datasets, together with clinical validation, demonstrated that ADAR1 is preferentially upregulated in malignant plasma cells and is associated with disease progression, poor prognosis, and reduced CD8^+^ T-cell infiltration. These findings suggest that ADAR1 contributes to the establishment of an immunosuppressive tumor microenvironment and prompted further investigation of its underlying mechanism.

ADAR1 is a central regulator of innate immune homeostasis through adenosine-to-inosine (A-to-I) RNA editing. By editing endogenous double-stranded RNA (dsRNA), ADAR1 prevents inappropriate activation of innate immune sensors, including MDA5 and PKR, thereby maintaining immune tolerance. Dysregulated ADAR1 expression has been implicated in immune evasion, tumor progression, and resistance to immune checkpoint blockade in multiple solid tumors, including melanoma, lung cancer, breast cancer, and cervical cancer [14,20,21,22]. Previous studies have shown that genetic deletion or pharmacological targeting of ADAR1 restores endogenous dsRNA sensing, activates type I interferon signaling, enhances immune cell infiltration, and sensitizes tumors to immune checkpoint blockade [23,24]. However, the functional significance and mechanistic basis of ADAR1-mediated immune regulation in MM have remained largely unexplored.

Our findings extend these observations to MM and define an ADAR1–MDA5-type I interferon signaling axis that links RNA editing to tumor-intrinsic immune suppression. RNA immunoprecipitation assays demonstrated that ADAR1 knockdown increased the association of endogenous Alu dsRNA with MDA5, providing direct evidence that ADAR1-mediated RNA editing limits innate immune recognition of endogenous dsRNA. Consequently, restoration of MDA5 signaling enhanced IFNα production and downstream STAT1 activation, leading to increased CD8^+^ T-cell proliferation and cytotoxicity. Importantly, these effects were largely abolished by MDA5 depletion, establishing MDA5 as the critical mediator of ADAR1-dependent immune regulation in MM. Together, these findings establish RNA editing as a key mechanism through which malignant plasma cells suppress innate immune sensing and antitumor immunity.

The bone marrow microenvironment is an important determinant of immune function in MM. Bone marrow stromal cells regulate cytokine production and influence both tumor cell survival and immune responses. To distinguish tumor-intrinsic effects from microenvironmental regulation, we included stromal cell-free controls in our co-culture experiments. Although HS-5 cells amplified IFNα production and more closely recapitulated the bone marrow microenvironment, ADAR1 depletion alone was sufficient to activate type I interferon signaling, enhance CD8^+^ T-cell function, and suppress myeloma cell growth. These findings indicate that activation of the ADAR1–MDA5–IFNα axis is primarily driven by tumor cell-intrinsic mechanisms, while stromal cells further potentiate this response.

From a translational perspective, our findings support targeting the ADAR1 pathway to enhance immunotherapy in MM. In an immunocompetent mouse model, treatment with 8-azaadenosine, particularly in combination with PD-1 blockade, significantly suppressed tumor growth, increased tumor cell apoptosis, enhanced IFNα expression, and promoted intratumoral CD8^+^ T-cell infiltration. These findings are consistent with accumulating evidence that restoration of endogenous dsRNA sensing can overcome immune evasion and improve the efficacy of immune checkpoint blockade [14,20,25]. Although 8-azaadenosine is not a fully specific ADAR1 inhibitor, the concordance between the pharmacological and genetic loss-of-function experiments supports the therapeutic potential of targeting the ADAR1 pathway in MM.

Several limitations should be acknowledged. Although our data establish a functional link between ADAR1 and MDA5-dependent type I interferon signaling, the specific RNA-editing events responsible for immune activation and the upstream mechanisms regulating ADAR1 expression in MM remain to be elucidated. In addition, the clinical cohort was relatively small, and larger independent studies are needed to validate the diagnostic and prognostic significance of ADAR1. Finally, while 8-azaadenosine was used to target the ADAR1 pathway in vivo, previous studies have suggested that it lacks complete specificity and may exert off-target effects. Therefore, the observed antitumor activity cannot be attributed solely to ADAR1 inhibition. Nevertheless, the major mechanistic conclusions were consistently supported by ADAR1 knockdown and RNA immunoprecipitation experiments. Future studies using more selective ADAR1 inhibitors or genetically engineered mouse models are warranted to further define the therapeutic value of targeting ADAR1 in MM.

**Figure 8:**
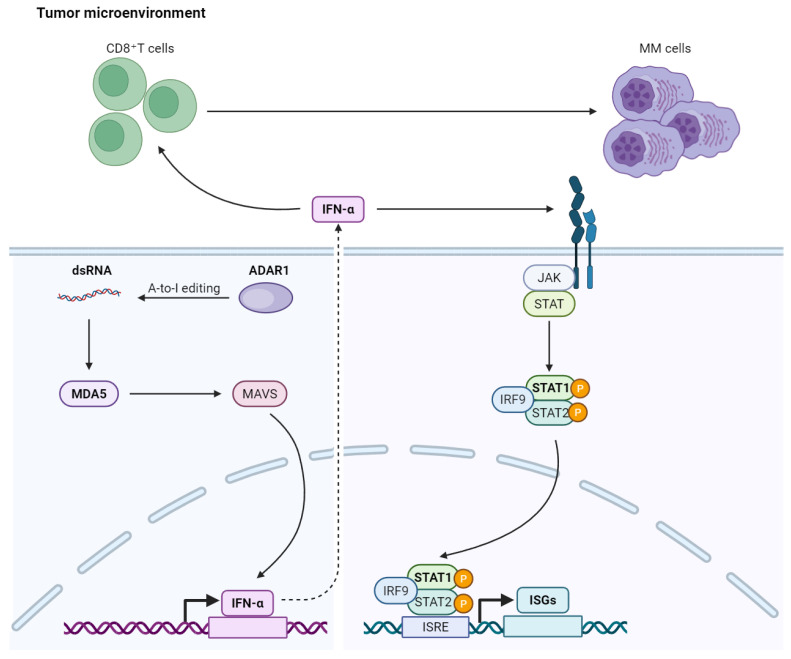
Proposed model of ADAR1-mediated immune evasion in multiple myeloma. ADAR1 promotes A-to-I editing of endogenous dsRNA, thereby limiting its recognition by the innate immune sensor MDA5. Suppression of the MDA5–MAVS pathway attenuates type I interferon signaling, resulting in reduced IFN-α production and impaired downstream JAK/STAT activation. Consequently, CD8^+^ T-cell function is compromised, contributing to the establishment of an immunosuppressive bone marrow microenvironment.

In summary, this study identifies ADAR1 as a critical tumor-intrinsic regulator of immune evasion in MM and demonstrates that ADAR1-mediated RNA editing suppresses MDA5-dependent recognition of endogenous dsRNA, thereby attenuating type I interferon signaling and impairing CD8^+^ T-cell-mediated antitumor immunity. Targeting the ADAR1–MDA5-type I interferon axis restores innate immune activation and enhances the efficacy of PD-1 blockade, providing a mechanistic rationale for combination immunotherapy in MM. Based on these findings, we propose a working model illustrating how ADAR1-mediated RNA editing promotes immune escape through suppression of MDA5-dependent innate immune signaling (Figure 8).

## 4. Materials and Methods

### 4.1. Data Acquisition and Preprocessing

Bulk transcriptomic and single-cell RNA sequencing datasets were obtained from the Gene Expression Omnibus (GEO) database. Raw expression matrices, platform annotation files, and corresponding clinical data were downloaded using the GEOquery R package (version 2.76.0). For microarray datasets, probe identifiers were mapped to gene symbols according to platform annotations, and the average expression value was calculated when multiple probes corresponded to the same gene. Expression matrices were log2-transformed and quantile-normalized prior to downstream analyses. Samples lacking clinical or follow-up information were excluded. In all analyses, “ADAR” specifically refers to ADAR1.

### 4.2. Immune Infiltration Analysis

The relative abundance of immune cell populations was estimated using the CIBERSORT algorithm with the LM22 signature matrix, which quantifies 6 main immune cell subsets from bulk transcriptomic data via support vector regression. Immune infiltration patterns were first compared between MM and healthy donor bone marrow samples in the GSE39754 dataset. Associations between immune cell composition and OS were subsequently evaluated in the GSE24080 cohort. To assess the relationship between ADAR1 expression and immune infiltration, samples in GSE39754 were stratified into ADAR1-high and ADAR1-low groups based on the median expression level.

### 4.3. Identification of Candidate Genes Associated with Immune Dysfunction

Differentially expressed genes (DEGs) between MM and healthy samples were identified in the GSE39754 dataset using the limma R package (version 3.64.3). Genes associated with CD8^+^ T-cell infiltration were determined by Spearman correlation analysis between gene expression and the estimated proportion of CD8^+^ T cells. Prognosis-related genes were identified using univariate Cox regression analysis in the GSE4204 dataset. Candidate genes involved in immune regulation were defined as the intersection of DEGs, CD8^+^ T-cell–associated genes, and survival-related genes.

### 4.4. Clinical Relevance of ADAR1 Expression

ADAR1 expression levels in bone marrow samples from MM samples and healthy samples were evaluated using GSE39754 and GSE6477 datasets. Receiver operating characteristic (ROC) curve analysis was performed using the pROC R package (version 1.18.5) to assess diagnostic performance, and the area under the curve (AUC) was calculated. Kaplan–Meier survival analysis was conducted to evaluate the association between ADAR1 expression and OS, with statistical significance determined using the log-rank test. Patients were stratified into ADAR1-high and ADAR1-low groups based on the median expression level.

### 4.5. Immune Correlation Network Analysis

Spearman correlation coefficients were calculated among 6 main immune cell subsets estimated by CIBERSORT in the GSE39754 dataset to construct an immune correlation network. The relationships between ADAR1 expression and the infiltration of individual immune cell types were further analyzed, with particular focus on CD8^+^ T cells. Corre-lation patterns were visualized using heatmaps and scatter plots.

### 4.6. Single-Cell Transcriptomic Analysis

Single-cell RNA sequencing data from the GSE124310 dataset were processed using the Seurat workflow, including normalization, dimensionality reduction, and clustering. Uniform manifold approximation and projection (UMAP) was applied for visualization, and cell types were annotated based on canonical markers. ADAR1 expression across cell populations was visualized using FeaturePlot and VlnPlot functions implemented in the Seurat R package (version 5.5.0). Plasma cell clusters were further extracted to compare ADAR1 expression between MM-derived and healthy donor plasma cells.

### 4.7. Pathway Activity and T-Cell Exhaustion Analysis

Plasma cell subsets were stratified into ADAR1-high and ADAR1-low groups based on median expression. Type I IFN pathway activity was quantified using the AddModuleScore function in Seurat (version 5.5.0), based on a predefined gene set (IFIT1, IFIT3, ISG15, MX1, OAS1, and STAT1). T-cell subsets were extracted to evaluate exhaustion signatures using established markers (PDCD1, LAG3, HAVCR2, TIGIT, and CTLA4). Differences between MM and healthy samples were subsequently assessed.

### 4.8. RNA Editing Activity Analysis

RNA editing activity was evaluated using the GSE110486 dataset. A-to-G editing frequencies were compared between ADAR1 wild-type and mutant MM1s cells, as well as between ADAR1 knockdown U266 cells (shADAR1 #1/#3) and corresponding controls (EV or shCtrl). Relative changes in editing activity were calculated as the percentage difference between experimental and control groups.

### 4.9. Clinical Samples and Ethical Approval

Bone marrow and peripheral blood samples were collected from 40 patients with newly diagnosed MM and 20 healthy donors between November 2024 and October 2025 at the Department of Hematology, The Second Affiliated Hospital of Harbin Medical University. MM diagnosis was established according to International Myeloma Working Group criteria. Samples were used for quantitative real-time PCR (qRT-PCR), ELISA, and immunohistochemistry (IHC) analyses. All participants provided written informed consent. The study was approved by the Ethics Committee of the Second Affiliated Hospital of Harbin Medical University (Approval No. KY2024-169) and was conducted in accordance with the Declaration of Helsinki.

### 4.10. PBMC Isolation and Quantitative Real-Time PCR

Peripheral blood mononuclear cells (PBMCs) were isolated from EDTA-anticoagulated blood using Ficoll-Paque PLUS density gradient centrifugation. Total RNA was extracted using TRIzol reagent (15596026CN, ThermoFisher, Waltham, MA, USA) and quantified using a NanoDrop spectrophotometer (BioSpec-nano, 206-26300-48, Shimadzu Corporation, Kyoto, Japan). cDNA synthesis was performed by reverse transcription, followed by qRT-PCR using a SYBR Green detection system. GAPDH was used as an internal control, and relative expression levels were calculated using the 2^−ΔΔCt^ method. Primer sequences were as follows: ADAR1, forward 5′-TGGGCATTAGGTTCTCCAGC-3′ and reverse 5′-CAGCAGATGCGTGGAATTCAG-3′; GAPDH, forward 5′-CATGTTCGTCATGGGTGTGAA-3′ and reverse 5′-GGCATGGACTGTGGTCATGAG-3′.

### 4.11. Immunohistochemistry

Paraffin-embedded human and mouse tumor tissue sections (4 μm) were deparaffinized, rehydrated, and subjected to antigen retrieval using citrate buffer (pH 6.0). Endogenous peroxidase activity was blocked with 3% hydrogen peroxide. For human samples, sections were incubated overnight at 4 °C with primary antibodies against CD8 (CY5219), CD45 (CY5311), and NCAM1/CD56 (CY6683) from Abways (Shanghai, China). For mouse tumor tissues, CD8 staining was performed using an anti-CD8 antibody (AF5126) from Affinity (Changzhou, China). After incubation, sections were incubated with HRP-conjugated secondary antibodies. Signals were visualized using 3,3′-diaminobenzidine (DAB), and nuclei were counterstained with hematoxylin.

### 4.12. Cell Lines and Culture Conditions

Human MM cell lines U266 (iCell-h266) and H929 (iCell-h311), as well as the bone marrow stromal cell line HS-5 (iCell-h436), were obtained from Cellverse (Shanghai, China). The murine MM cell line MOPC315 (TCM-C797) was purchased from Haixing (Suzhou, China). U266 and H929 cells were cultured in RPMI-1640 medium supplemented with 10% fetal bovine serum (FBS) and 1% penicillin–streptomycin, with β-mercaptoethanol (0.05 mM) added for H929 cells. HS-5 cells were maintained in DMEM supplemented with 10% FBS and 1% penicillin–streptomycin. All cells were cultured at 37 °C in a humidified incubator with 5% CO_2_.

### 4.13. Isolation and Expansion of CD8^+^ T Cells

Primary human CD8^+^ T cells were isolated from healthy donor PBMCs using magnetic bead-based sorting. Purified cells were activated and expanded using a cytotoxic T-cell expansion kit (CT-006, Stemery, Xiamen, China) according to the manufacturer’s protocol to obtain sufficient functional cells for downstream assays.

### 4.14. Co-Culture Systems

A non-contact co-culture system was established using 0.4 μm Transwell inserts, with HS-5 stromal cells seeded in the upper chamber and U266 or H929 myeloma cells cultured in the lower chamber at a 2:1 ratio for 48 h. This dual-culture system was used to assess IFNα/IFNβ production and signaling pathway activation.

To simulate tumor–stromal–immune interactions, CD8^+^ T cells were subsequently added to form a triple co-culture system (U266/H929:HS-5:CD8^+^ T = 2:1:2), followed by an additional 48 h incubation. This configuration was employed for evaluating T-cell proliferation, cytotoxicity, myeloma cell proliferation, and apoptosis. Functional assays within this triple co-culture system included the following experimental conditions: (i) ADAR1 knockdown (si-ADAR1) with HS-5 and CD8^+^ T cells; (ii) ADAR1 and MDA5 double knockdown (si-ADAR1-MDA5) with HS-5 and CD8^+^ T cells; (iii) ADAR1 and STAT1 double knockdown (si-ADAR1-STAT1) with HS-5 and CD8^+^ T cells; (iv) negative-control siRNA-transfected myeloma cells with HS-5 and CD8^+^ T cells (NC); (v) NC co-cultures supplemented with recombinant human IFNα (10 ng/mL, NC+IFNα); and (vi) MM cells with CD8^+^ T cells in the absence of HS-5 cells (NC-).

### 4.15. siRNA Transfection

Small interfering RNAs (siRNAs) targeting ADAR1, MDA5, and STAT1, as well as a negative control siRNA (si-NC), were transfected into U266 and H929 cells using CALNP™ RNAi transfection reagent (DN001, D-Nano, Beijing, China) according to the manufacturer’s instructions. Cells were transfected during the logarithmic growth phase, and knockdown efficiency was assessed by qRT-PCR 24–48 h post-transfection. The siRNA sequences were as follows: si-ADAR1, sense 5′-CGGAUACUACACCCAUCCAUU-3′ and antisense 5′-AAUGGAUGGGUGUAGUAUCCG-3′; si-MDA5, sense 5′-GAGAGAAGAUGAUGUAUAA-3′ and antisense 5′-UUAUACAUCAUCUUCUCUC-3′; si-STAT1, sense 5′-GAACAGAAAUACACCUACGAA-3′ and antisense 5′-UUCGUAGGUGUAUUUCUGUUC-3′; and si-NC, sense 5′-UUCUCCGAACGUGUCACGUTT-3′ and antisense 5′-ACGUGACACGUUCGGAGAATT-3′. The primer sequences used for knockdown validation were as follows: ADAR1, forward 5′-TGGGCATTAGGTTCTCCAGC-3′ and reverse 5′-CAGCAGATGCGTGGAATTCAG-3′; MDA5, forward 5′-GCTGAAGTAGGAGTCAAAGCCC-3′ and reverse 5′-CCACTGTGGTAGCGATAAGCAG-3′; STAT1, forward 5′-ATGGCAGTCTGGCGGCTGAATT-3′ and reverse 5′-CCAAACCAGGCTGGCACAATTG-3′; and GAPDH, forward 5′-CATGTTCGTCATGGGTGTGAA-3′ and reverse 5′-GGCATGGACTGTGGTCATGAG-3′.

### 4.16. IFNα Stimulation and PD-1 Blockade

For IFN stimulation, recombinant human IFNα (10 ng/mL; MedChemExpress, HY-P78672, Monmouth Junction, NJ, USA) was added to the co-culture system and incubated for 48 h. For immune checkpoint inhibition, pembrolizumab (20 μg/mL; MedChemExpress, Cat. No. HY-P9902) was applied for 48 h. Control groups received PBS or isotype control antibodies.

### 4.17. RIP Assay

RIP assays were performed using a RIP kit (Beyotime, P1801S) (P1801S, Beyotime Biotechnology, Shanghai, China) according to the manufacturer’s instructions. MM cells were transfected with control siRNA (NC) or si-ADAR1 and lysed in RIP lysis buffer supplemented with RNase and protease inhibitors.

Cell lysates were incubated overnight at 4 °C with anti-MDA5 antibody (21775-1-AP) or rabbit IgG control (30000-0-AP) (Proteintech Group, Wuhan, China), followed by capture with Protein A/G agarose beads. After extensive washing, a fraction of immunoprecipitates was used for Western blot to confirm MDA5 enrichment.

Co-precipitated RNA and input were extracted using TRIzol (15596026CN, ThermoFisher, Waltham, MA, USA), reverse transcribed, and analyzed by qRT-PCR to assess enrichment of Alu and HERV-K transcripts. Primer sequences were as follows: Alu consensus, Forward 5′-CACGCCTGTAATCCCAGCACTTT-3′ and Reverse 5′-CCCAGGCTGGAGTGCAGTGG-3′; HERV-K, Forward 5′-TGTGTGCTGACTCAGGAGGA-3′ and Reverse 5′-CCTCCTGTTCTCTCTTCTGC-3′.

### 4.18. Western Blot Analysis

Total protein was extracted using RIPA lysis buffer, and concentrations were determined by BCA assay. Proteins from cultured cells and mouse tumor tissues were separated by SDS-PAGE and transferred onto PVDF membranes. After blocking, membranes were incubated overnight at 4 °C with primary antibodies against STAT1 (DF6086), ISG15 (DF6316), IFNα (DF6086), and IFNβ (DF6471) from Affinity (Changzhou, China), and GAPDH (AB0037) from Abways (Shanghai, China), followed by HRP-conjugated secondary antibodies. Signals were detected using enhanced chemiluminescence.

### 4.19. ELISA

Levels of IFNα, IFNβ, perforin (PF1), and granzyme B (GZMB) in culture supernatants were quantified using ELISA kits according to the manufacturer’s instructions: human IFNα2 (JL31563-96T), human IFNβ (JL19215-96T), human perforin 1 (JL23588-96T), and human granzyme B (JL10402-96T) from JONLNBIO (Shanghai, China).

### 4.20. Cell Proliferation and Apoptosis Assays

Cell proliferation was assessed using the CCK-8 assay. Cells were seeded in 96-well plates (1 × 10^5^ cells per well) and incubated with CCK-8 reagent for 2 h before measuring absorbance at 450 nm. Apoptosis was evaluated using Annexin V-FITC/PI staining (SC123, Beijing, China) followed by flow cytometry analysis with FlowJo software (version 10.8.1).

### 4.21. TUNEL Assay

Apoptosis in tumor tissues was assessed using a TUNEL Cell Apoptosis Assay Kit (C1179, Beyotime, Shanghai, China) according to the manufacturer’s instructions. Paraffin-embedded tumor sections were deparaffinized, rehydrated, and treated with DNase-free proteinase K (20 μg/mL). Sections were then incubated with EdUTP labeling solution and Click reaction solution, followed by Hoechst 33342 counterstaining provided in the kit. Fluorescent images were captured using a fluorescence microscope.

### 4.22. Animal Experiments

Female BALB/c mice (6–8 weeks old) were used to establish a subcutaneous MOPC315 tumor model. Mice were housed in a specific pathogen-free facility under controlled conditions (22 ± 2 °C, 12 h light/dark cycle) with ad libitum access to food and water. When tumor volumes reached 80–100 mm^3^, mice were randomized into four groups (n = 5 per group): control, ADAR1 inhibitor (8-azaadenosine; HY-115686), PD-1 blockade (anti-mouse PD-1 antibody; HY-P99144), and combination therapy (MedChemExpress, Monmouth Junction, NJ, USA). Treatments were administered intraperitoneally every three days for three cycles (2.5 mg/kg each). Tumor size was measured every three days.

Mice were monitored daily for signs of distress, including weight loss exceeding 20% or tumor ulceration. Mice were euthanized when tumor volume exceeded 1500 mm^3^, reached predefined humane endpoints, or at the end of the study. Euthanasia was performed by cervical dislocation. All animal procedures were approved by the Animal Ethics Committee of the Second Affiliated Hospital of Harbin Medical University (Approval No. YJSDW2024-198).

### 4.23. Statistical Analysis

Statistical analyses were performed using SPSS 26.0. Continuous variables were presented as mean ± standard deviation or median (interquartile range). Differences between two groups were assessed using Student’s *t*-test or the Mann–Whitney U test, while multiple group comparisons were performed using one-way ANOVA with Tukey’s post hoc test or the Kruskal–Wallis test with Dunn’s correction. Correlations were evaluated using Spearman analysis. Survival analysis was conducted using the Kaplan–Meier method with the log-rank test. Cox regression analysis was used to identify prognostic factors. ROC analysis was performed using the pROC package (version 1.18.5). Multiple testing correction was applied using the Benjamini–Hochberg method.

## 5. Conclusions

This study identifies ADAR1 as a previously unappreciated tumor-intrinsic brake on anti-myeloma immunity. Elevated ADAR1 in malignant plasma cells suppresses MDA5-dependent type I interferon sensing, blunting IFNα production and STAT1 activation while limiting CD8^+^ T-cell recruitment and function. Disrupting this axis—either by ADAR1 silencing or pharmacological inhibition—restores interferon signaling, reinvigorates cytotoxic T-cell activity, and overcomes resistance to PD-1 blockade. These findings position ADAR1 as a central link between RNA editing and immune evasion in multiple myeloma and support its therapeutic targeting to improve responses to immunotherapy.

## Data Availability

The data presented in this study are openly available in Gene Expression Omnibus at https://www.ncbi.nlm.nih.gov/geo/, reference numbers GSE39754, GSE24080, GSE4204, GSE6477, GSE124310, and GSE110486.

## Abbreviations

The following abbreviations are used in this manuscript:

ADAR1	Adenosine deaminase acting on RNA 1
MM	Multiple myeloma
BM	Bone marrow
PB	Peripheral blood
NK	Natural killer
MDA5	Melanoma differentiation–associated protein 5
CTLs	CD8^+^ cytotoxic T lymphocytes
IFN	Interferon
Tregs	Regulatory T cells
GEO	Gene Expression Omnibus
OS	Overall survival
DEGs	Differentially expressed genes
ROC	Receiver operating characteristic
AUC	Area under the curve
UMAP	Uniform manifold approximation and projection
qRT-PCR	Quantitative real-time PCR
PBMCs	Peripheral blood mononuclear cells
FBS	Fetal bovine serum
RIP	RNA immunoprecipitation
GZMB	Granzyme B
PF1	Perforin 1

## References

[1] Cowan A.J., Green D.J., Kwok M., Lee S., Coffey D.G., Holmberg L.A., Tuazon S., Gopal A.K., Libby E.N. (2022). Diagnosis and Management of Multiple Myeloma: A Review. JAMA.

[2] Rafae A., van Rhee F., Al Hadidi S. (2024). Perspectives on the Treatment of Multiple Myeloma. Oncologist.

[3] Lu Q., Yang D., Li H., Niu T., Tong A. (2024). Multiple myeloma: Signaling pathways and targeted therapy. Mol. Biomed..

[4] Garfall A.L. (2024). New Biological Therapies for Multiple Myeloma. Annu. Rev. Med..

[5] Sharma N.S., Choudhary B. (2023). Good Cop, Bad Cop: Profiling the Immune Landscape in Multiple Myeloma. Biomolecules.

[6] Cheng Y., Sun F., Alapat D.V., Wanchai V., Mery D., Siegel E.R., Xu H., Johnson S., Guo W., Bailey C. (2024). Multi-omics reveal immune microenvironment alterations in multiple myeloma and its precursor stages. Blood Cancer J..

[7] Wan Y., Wang J., Chen M., Wang J., Nan F., Huang H., Liu Z., Hou J. (2025). Dual roles of IRE1α inhibition in reversing mitochondrial ROS-induced CD8^+^ T-cell senescence and exerting direct antitumor effects in multiple myeloma. J. Immunother. Cancer.

[8] Ma J., Gao S., Liu S., Chen L., Lin L., Zhang Z., Zhao Z., Li Q. (2026). Single-cell profiling reveals MIF-mediated immune evasion and CD8^+^ T cell exhaustion in relapsed multiple myeloma. Clin. Exp. Med..

[9] Frede J., Poller J.C., Shi K., Stuart H., Sotudeh N., Havig C., Lim K., Wiggers C.R.M., Cho E.Y., Vijaykumar T. (2025). The endogenous T cell landscape is reshaped by CAR-T cell therapy and predicts treatment response in multiple myeloma. Leukemia.

[10] Shi Y., McKenery A., Dolan M., Mastri M., Hill J.W., Dommer A., Benzekry S., Long M., Abrams S.I., Puzanov I. (2025). Acquired resistance to PD-L1 inhibition enhances a type I IFN-regulated secretory program in tumors. EMBO Rep..

[11] Kalbasi A., Tariveranmoshabad M., Hakimi K., Kremer S., Campbell K.M., Funes J.M., Vega-Crespo A., Parisi G., Champekar A., Nguyen C. (2020). Uncoupling interferon signaling and antigen presentation to overcome immunotherapy resistance due to JAK1 loss in melanoma. Sci. Transl. Med..

[12] Sun T., Li Q., Geisinger J.M., Hu S.B., Fan B., Su S., Tsui W., Guo H., Ma J., Li J.B. (2025). ADAR1 editing is necessary for only a small subset of cytosolic dsRNAs to evade MDA5-mediated autoimmunity. Nat. Genet..

[13] Miller C.M., Morrison J.H., Bankers L., Dran R., Kendrick J.M., Briggs E., Ferguson V.L., Poeschla E.M. (2025). ADAR1 haploinsufficiency and sustained picornaviral RdRp dsRNA synthesis synergize to dysregulate RNA editing and cause multi-system interferonopathy. mBio.

[14] Jia W., Ma L., Bai M., Li J., Jin P., Wang M., Li J., Guo X., Tian Y., Guo H. (2025). Loss of ADAR1 in lung cancer activates anti-tumour immunity and suppresses tumour cell growth via the RIG-I/MDA5-MAVS pathway. Cancer Lett..

[15] Mohty M., Facon T., Malard F., Harousseau J.L. (2024). A roadmap towards improving outcomes in multiple myeloma. Blood Cancer J..

[16] Bhatt P., Kloock C., Comenzo R. (2023). Relapsed/Refractory Multiple Myeloma: A Review of Available Therapies and Clinical Scenarios Encountered in Myeloma Relapse. Curr. Oncol..

[17] Iannozzi N.T., Giuliani N., Storti P. (2025). Deciphering the bone marrow microenvironment’s role in multiple myeloma immunotherapy resistance. Front. Immunol..

[18] Foster K.A., Rees E., Ainley L., Laidler A., Boyle E.M., Lee L., Ward G., Galas-Filipowicz D., Fitzsimons E., Mikolajczak A. (2026). Tumour-intrinsic features shape T cell differentiation through precursor to symptomatic multiple myeloma. Nat. Commun..

[19] Shasha C., Glass D.R., Moelhman E., Islas L., Tian Y., Chour T., Xu G., Szeto G.L., Peng T., Song X. (2025). Hallmarks of T-cell exhaustion and antigen experience are absent in multiple myeloma from diagnosis to maintenance therapy. Blood.

[20] Ishizuka J.J., Manguso R.T., Cheruiyot C.K., Bi K., Panda A., Iracheta-Vellve A., Miller B.C., Du P.P., Yates K.B., Dubrot J. (2019). Loss of ADAR1 in tumours overcomes resistance to immune checkpoint blockade. Nature.

[21] Chen S.Y., Chen S.Y., Yang S., Li Y., Yang S.Y. (2025). ADAR1-mediated RNA editing in breast cancer: Molecular mechanisms and therapeutic implications. Med. Oncol..

[22] Nakamura K., Shigeyasu K., Okamoto K., Matsuoka H., Masuyama H. (2023). ADAR1 has an oncogenic function and can be a prognostic factor in cervical cancer. Sci. Rep..

[23] Zhang T., Yin C., Fedorov A., Qiao L., Bao H., Beknazarov N., Wang S., Gautam A., Williams R.M., Crawford J.C. (2022). ADAR1 masks the cancer immunotherapeutic promise of ZBP1-driven necroptosis. Nature.

[24] Gannon H.S., Zou T., Kiessling M.K., Gao G.F., Cai D., Choi P.S., Ivan A.P., Buchumenski I., Berger A.C., Goldstein J.T. (2018). Identification of ADAR1 adenosine deaminase dependency in a subset of cancer cells. Nat. Commun..

[25] Tassinari V., Kaciulis M., Petrai S., Stabile H., Pernazza A., Leopizzi M., Di Maio V., Belleudi F., Ranieri D., Mancini V. (2025). ADAR1 expression is associated with cervical cancer progression and negatively regulates NK cell activity. JCI Insight.

